# Quantifying Everyday Ecologies: Principles for Manual Annotation of Many Hours of Infants' Lives

**DOI:** 10.3389/fpsyg.2021.710636

**Published:** 2021-09-06

**Authors:** Jennifer K. Mendoza, Caitlin M. Fausey

**Affiliations:** Department of Psychology, University of Oregon, Eugene, OR, United States

**Keywords:** annotation, input, music, infancy, LENA

## Abstract

Everyday experiences are the experiences available to shape developmental change. Remarkable advances in devices used to record infants' and toddlers' everyday experiences, as well as in repositories to aggregate and share such recordings across teams of theorists, have yielded a potential gold mine of insights to spur next-generation theories of experience-dependent change. Making full use of these advances, however, currently requires manual annotation. Manually annotating many hours of everyday life is a dedicated pursuit requiring significant time and resources, and in many domains is an endeavor currently lacking foundational facts to guide potentially consequential implementation decisions. These realities make manual annotation a frequent barrier to discoveries, as theorists instead opt for narrower scoped activities. Here, we provide theorists with a framework for manually annotating many hours of everyday life designed to reduce both theoretical and practical overwhelm. We share insights based on our team's recent adventures in the previously uncharted territory of everyday music. We identify principles, and share implementation examples and tools, to help theorists achieve scalable solutions to challenges that are especially fierce when annotating extended timescales. These principles for quantifying everyday ecologies will help theorists collectively maximize return on investment in databases of everyday recordings and will enable a broad community of scholars—across institutions, skillsets, experiences, and working environments—to make discoveries about the experiences upon which development may depend.

## Introduction

Experience-dependent changes in neural circuitry and behavior are central to development (Hensch, 2005; Hannon and Trainor, 2007; Scott et al., 2007; Aslin, 2017). Complete theories of development must therefore model the experiences that drive change. In human infancy, detailed models of real-world early experiences have traditionally been hard to achieve because of challenges in recording everyday sensory histories. Recent technological advances that permit many hours of recording have minimized this barrier of experience sampling *per se* (de Barbaro, 2019). One insight from recent efforts using these technologies is that when relevant sensory histories naturally unfold over extended timescales, shorter samples miss pervasive properties of infants' everyday ecologies. For example, short language samples miss typical rhythms of interleaving speech and silence (Tamis-LeMonda et al., 2017; Cristia et al., 2021) and short musical samples (Mendoza and Fausey, 2021a) fail to capture opportunities for repetition and variability as instances arise non-uniformly over time (Smith et al., 2018). An emerging priority for theories of development is therefore to model very large amounts of everyday experience. Though recording such quantities is now possible, automatically detecting relevant units within the complex and varied sensory streams of everyday life is not (Adolph, 2020; de Barbaro and Fausey, in press). Developmental theorists must therefore tackle the challenge of manually annotating many hours of everyday life.

Manually annotating many hours of everyday life is so daunting that most researchers who have recorded such data avoid it. The status quo is to declare longform manual annotation “impractical,” “untenable,” “not realistic,” “challenging,” and “unwieldy” (Roy et al., 2015; Casillas et al., 2017; Tamis-LeMonda et al., 2018; Casillas and Cristia, 2019; Räsänen et al., 2019). Despite developmental theorists' considerable expertise in annotating behavior (Bakeman and Gottman, 1997; Adolph, 2020), scaling from researcher-constrained short activities to everyday ecologies is not straightforward. One challenge is that everyday sights and sounds are not just “more” data, but also “different” data. Theorists must update operationalizations of annotation targets based on new and variable instantiations arising in everyday sensory streams. Another challenge is a lower signal-to-noise ratio in everyday contexts compared to researcher-constrained contexts because of multiple overlapping sources generating the sensory streams. Audio data, in particular, are often literally “noisier” (Xu et al., 2009). Reaching conventional thresholds for reliably identifying annotation targets is therefore a Sisyphean task that often demands updated rationale. Finally, because successful annotation requires manyfold the duration of the annotated recording (MacWhinney, 2000), manually annotating many hours of everyday life requires very large investments of time, personnel, and dedicated resources including sustained funding (Casillas and Cristia, 2019; VanDam and De Palma, 2019). Theorists must achieve remarkable “operations manager” prowess in their laboratories. This suite of challenges is fierce but it need not thwart research progress. Here, we articulate principles for manually annotating many hours of everyday life that minimize challenges and maximize opportunities for new discoveries about infants' everyday ecologies.

The potential for new discoveries about infants' everyday ecologies is perhaps higher than ever, given repositories of everyday experiences like Databrary (https://nyu.databrary.org/; Gilmore et al., 2018) and HomeBank (https://homebank.talkbank.org/; VanDam et al., 2016). Each of these repositories already contains many hours of recordings captured from infants' everyday lives that are available for theorists to annotate. Regularities in everyday audio, including multiple levels of vocalization, language, and music, as well patterns in multi-modal video including emotional expressions, contingencies and motor dynamics among social partners, and nameable object and actions, are hypothesized to shape developmental change. Thus, quantifying these everyday regularities will inform developmental theory including computational models that currently lack everyday parameters.

As a scientific process, manual annotation of many hours of everyday life is also well-suited to priorities like expanding our scientific workforce by including people, expertises, and institutions who have traditionally faced systemic barriers to participation in discovery. For example, though not every investigator may always have resources to innovate technology or to collect massive samples of new data, a very large number of scientists and their teams can conduct manual annotation of already existing everyday data. Further, the opportunity to aggregate across diverse samples of everyday data—each individual corpus in Datavyu and HomeBank is necessarily limited by space, time, and community—demands theorists' engagement in order to determine the extent to which findings vary across cultural contexts (Nielsen et al., 2017; Hruschka et al., 2018; Cychosz et al., 2020a; Soderstrom et al., 2021). Finally, as we experience disruptions like the COVID-19 pandemic and other barriers to traditional laboratory business-as-usual, manual annotation of many hours of everyday recordings is a scientific endeavor that is both feasible and likely to yield theory-relevant insights. Manual annotation is a classic bottleneck in maximizing returns on scientific investments, especially when initial study design and data collection generate very large datasets of continuous recordings of infants' everyday ecologies. Manually annotating these everyday data will yield theoretical insights as well as create goldstandard training and evaluation sets en route to eventual automated annotation (Bambach et al., 2016; Ossmy et al., 2018; Räsänen et al., 2019). Given our own team's recent adventures, we share here critical reflections on practices for manual annotation of many hours of everyday lives likely to advance developmental theory.

We share seven principles and materials to support their implementation (osf.io/eb9pw, henceforth “OSF”; Mendoza and Fausey, 2019) based on our recent discoveries about everyday music in infancy (Fausey and Mendoza, 2018a,b; Mendoza and Fausey, 2018, 2021a,b). Briefly, we audio recorded 35 full days-in-the-lives-of-infants and then identified the musical features, voices, and tunes available over the course of each day. Because music cannot yet be automatically detected in recordings of everyday life (Mehr et al., 2019), we pre-processed and then double-annotated roughly 270 hours of everyday audio. Among other findings, we discovered that infants encounter roughly 1 h of music per day, a quarter of which is live and vocal, with some musical tunes and voices preferentially available. So that other theorists can build on these discoveries, and so that scholars across domains can tackle manually annotating many hours of everyday life, here we present a framework for guiding the many decisions in a manual annotation workflow.

Music is an illustrative domain because there is very little extant evidence to inform decisions about manual annotation. The early days of a discovery process—the situation in which most theorists find themselves when first scaling to quantify everyday ecologies—present distinct challenges for justifying analytic decisions. All-day is an illustrative timescale because days constrain activities and their accompanying sensory details (Hofferth and Sandberg, 2001; Galland et al., 2012; Roy et al., 2015; Montag et al., 2018), 16-h audio days are feasible to record (Ford et al., 2008; Ganek and Eriks-Brophy, 2018), and yet few discoveries about everyday experiences have harnessed this extended timescale (though see Soderstrom and Wittebolle, 2013; Weisleder and Fernald, 2013; Roy et al., 2015 for related approaches). We add to a growing set of resources designed to support manual annotation, like the CHAT manual for transcribing language (talkbank.org; MacWhinney, 2000), Datavyu and ELAN for annotating and analyzing audio and/or video data (Wittenburg et al., 2006; Datavyu Team, 2014) and the DARCLE and ACLEW Annotation Schemes (Casillas et al., 2017; Soderstrom et al., 2021) for annotating speech in prioritized subsets of daylong audio recordings. We emphasize the conceptual and implementation needs associated with manually annotating many hours of everyday life.

**Figure 1:**
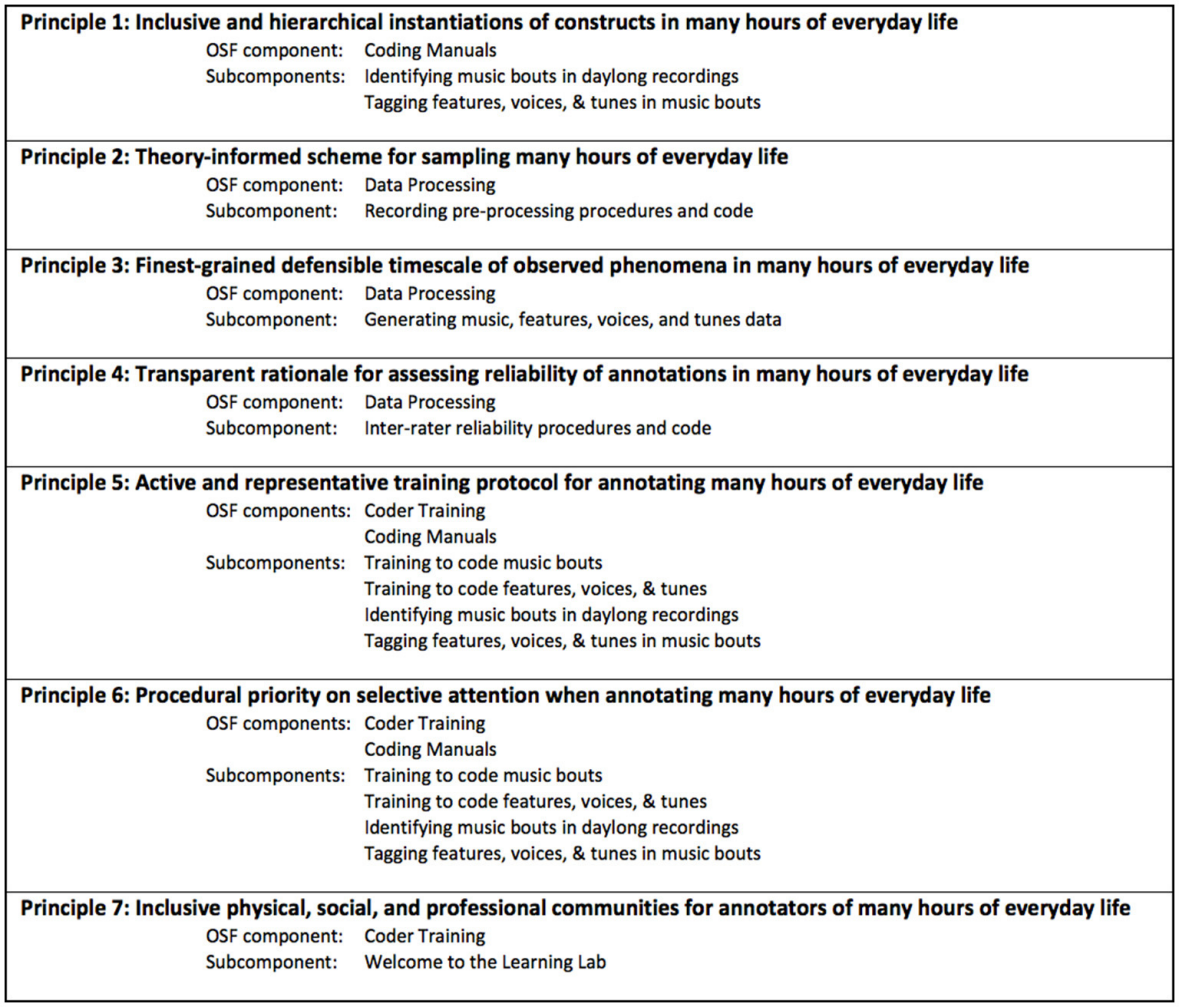
Principles for manual annotation of many hours of infants' everyday lives. Each principle guides decisions in a research program aiming to quantify everyday ecologies. Materials that support implementing these principles, instantiated in a line of research about infants' everyday music, are shared on OSF (https://osf.io/eb9pw/; Mendoza and Fausey, 2019). For an accessible version, please go to https://osf.io/vd8t5.

We share a set of principle-implementation pairs (Figure 1). We prioritize theorists' agency and so share a framework to structure decision-making rather than prescribing step-by-step instructions *per se*. Principles 1–3 address fundamental decisions about what to annotate in many hours of everyday life. Principles 4–6 address how to achieve reliable annotations at scale. Principle 7 addresses infrastructure for successful annotation. Each section of this paper presents one principle-implementation pair, first articulating the theoretical issues at stake and then describing implementation procedures. We share associated files like coding manuals and scripts on OSF to demystify the process and facilitate future efforts in the ambitious endeavor of making discoveries about infants' everyday ecologies.

## Principle 1: Inclusive and Hierarchical Instantiations of Constructs in Many Hours of Everyday Life

The core goal of manual annotation is to identify annotation targets within the stream of sensory experiences captured by a recording device. Because sensory histories are not uniform (e.g., Jayaraman et al., 2015; Roy et al., 2015; Tamis-LeMonda et al., 2017; Clerkin et al., 2018; Mendoza and Fausey, 2021a), annotation targets will sometimes be present and other times be absent throughout the recorded stream. Discovering structure in this everyday ecology requires identifying when instances of the “same” thing happen again even when separated in time, context, and with only partially overlapping instantiations. For example, over the course of a day, an infant might encounter a parent singing the first phrases of “Twinkle, Twinkle Little Star” at 8 a.m., a cartoon character singing “Wheels on the Bus” at 8:30 a.m., and then the same parent singing the entire “Twinkle, Twinkle Little Star” at 6 p.m. All of these example instances are music, all are vocal, some are live, some are recorded, and the voice and tune identities partially overlap across instances. Each kind of repetition and variation may be relevant for building musical skills like detecting multiple levels of musical structure, recognizing melodies, and generalizing musical meter (e.g., Hannon and Trehub, 2005; Margulis, 2014; Creel, 2019). Other potentially musical sounds may also arise in the infant's day, including some whistling, speech sound effects like “beep beep,” and clapping. Segmenting the stream of everyday experience so that repetitions and variations are discoverable requires detailed operationalizations of annotation targets.

The challenge when manually annotating many hours of everyday life is to achieve operationalizations that faithfully capture the everyday phenomena. Theorists often have little direct evidence from everyday life to guide decisions (one notable exception is language, in which “words” have long been recognized as important and transcribable units; MacWhinney, 2000). Extant evidence from researcher-constrained experiments often suggests relevant starting points. For example, musical sounds have been defined as “humanly produced, non-random sequences of tones or tone combinations that are non-referential” and vocal, instrumental, live, and recorded music have been instantiated in laboratory tasks (Trehub and Schellenberg, 1995). Infants also track repetition and variation across musical tunes and voices in these tasks (for review see Mendoza and Fausey, 2021a). Researcher-constrained instantiations of a construct are only starting points for operationalizing annotation targets because everyday sensory streams include a much wider range of activities, behaviors, and generating sources than laboratory contexts (Young and Gillen, 2007; Lamont, 2008; de Barbaro and Fausey, in press). For example, is vocal play from siblings (affectionately referred to as “scream singing” by our team) music? How about microwave beeping? Do humming and clapping deserve the same status as singing a complete rendition of “Happy Birthday”? Does half a rendition count? Many real-life instantiations of a construct have never been measured or manipulated in the laboratory (e.g., Lee et al., 2018). In the face of unknowns and potentially highly variable instantiations of to-be-annotated constructs over the course of many hours of everyday life, it is productive to operationalize a construct inclusively. Theorists can later quantify the prevalence and structure of specific subsets in future annotation efforts.

Multiple passes of manual annotation allow theorists to quantify a planned hierarchy of construct instantiations. For example, after inclusively annotating “music,” annotators can later identify features, voices, and tunes within the music, and then achieve even finer-grained transcriptions of pitches and rhythms. When very little about everyday ecologies is known, systematic manual annotation from more-general to less-general permits initial insights based on aggregating operationalizations across prior researcher-constrained investigations. This inclusive first pass thus identifies everyday structure that is broadly relevant to cumulative science. Insights about more specific instantiations (e.g., “live vocal complete renditions of tunes in C major”) may be difficult to initially discover given the sparsity of individual types in everyday ecologies (Zipf, 1936, 1949). If present, these instantiations can be quantified in subsequent annotation passes of inclusively annotated constructs.

There is no single instantiation of a construct across many hours of everyday life. There may not even be predictable deviations from a prototypical instantiation derived from prior evidence based on researcher-constrained tasks. The opportunity for discovery in everyday ecologies is real and so specific instantiations of constructs cannot always be entirely pre-planned. In order to operationalize inclusive and hierarchical instantiations of annotation targets in many hours of everyday life, theorists must therefore (1) conduct iterative pilot annotation, (2) create comprehensive definitions, and (3) find unambiguous examples for training.

### Implementation

We identified the onset and offset of individual instances of music (“music bouts”) in many hours of everyday life. Music bouts were operationalized inclusively. We then identified whether each music bout included live, recorded, vocal, and/or instrumental music. We also identified individual tune(s) and voice(s) in each music bout. After identifying the durations of music bouts, subsequent features were annotated as present or absent per bout given that durations would arise straightforwardly for those bouts containing a single feature (see Mendoza and Fausey, 2021a; we repeat some methodological rationale throughout). Figure 1 shows the OSF components that supported this annotation as we took the following steps in our workflow. One illustrative file is “3_FeaturesVoicesTunes_CodingManual.pptx” (https://osf.io/dtfnv/) which is the coding manual for identifying musical features, voices, and tunes in many hours of everyday life.

#### Iterative Pilot Annotation

Because many hours of everyday life present opportunities to discover previously unobserved instantiations of constructs, iterative “annotate-discuss-update” loops should inform eventual operationalizations that will guide the annotation of a planned sample of recordings. Theorists can avoid endless iterations by selecting pilot recordings from distinct family contexts and by engaging pilot annotators who have varying levels of expertise about children and the target domain. To develop our operationalizations for annotating everyday music, we completed several iterations of annotating recordings collected for this purpose. We solicited feedback from annotators about sounds that were easy or hard to identify as “music” in everyday ecologies. This process revealed the range of pitched and rhythmic sounds in a typical day of infants' lives. We discovered it was necessary to define not only the range of sounds that should be considered “music,” but also the range of sounds that should not be considered “music.” Our operationalizations were consistent with, and yet more specific and varied than, definitions in extant literature. We also annotated pilot recordings en route to everyday-appropriate operationalizations of musical features, voices, and tunes. Pilot annotators during this phase noted partial instantiations of standard tunes. For example, the pitch patterns in the first phrase (“Twinkle, twinkle little star”) and in the second phrase (“How I wonder what you are”) of “Twinkle, Twinkle Little Star” are different. If these phrases occurred in distinct musical bouts, would each bout receive the same tune annotation (“Twinkle, Twinkle Little Star”)? Reasonable theorists could arrive at different conclusions; we therefore emphasize making these decisions transparent by sharing detailed coding manuals and data so that future efforts can assess the extent to which such decisions matter for pattern discovery. Importantly, iterative pilot annotation revealed everyday instantiations of music and its features, voices, and tunes that were essential to address in annotation manuals.

#### Comprehensive Definitions

We arrived at a three-part definition of music that specified (1) the range of sounds that should be coded as music, (2) the range of sounds that should not be coded as music, and (3) the start and end of music bouts. We also clearly defined musical features (i.e., live, recorded, vocal, and/or instrumental music), voices, and tunes (Supplementary Table 2, OSF, https://osf.io/htx57/). In each definition, we highlighted the range of possible types that annotators might encounter. For example, our definition of vocal music lists several possible kinds of voices (e.g., adult, non-focal child, recorded character) and also the different types of vocal music (i.e., singing, humming, whistling, vocal play). We intentionally created definitions that emphasized the variability of instances that should be annotated.

#### Unambiguous Audio Examples for Training

In our two coding manuals, we combined the comprehensive definitions of music and musical properties with clear audio examples, extracted from pilot recordings, to illustrate each to-be-annotated phenomenon. We used audio examples that unambiguously depicted our phenomena. These prototypical anchors helped annotators decide what to do when they encountered an everyday sound in daylong audio recordings that was hard to annotate. For example, infants' older siblings commonly produced a very wide range of vocalizations, only some of which should be considered “music” under our annotation scheme. When an annotator encountered a sibling vocalization that they were not sure about, they could listen to the full set of audio examples that should be coded as “music” and the full set of audio examples that should not be coded as “music” and then decide to which set the specific sibling vocalization was most similar.

## Principle 2: Theory-Informed Scheme for Sampling Many Hours of Everyday Life

Theorists sample from everyday life when they record it and when they annotate it. Theorists must therefore choose how much and when to sample. The goal is to sample in such a way that allows theorists to make discoveries about everyday ecologies that both respect things we already know and move us in some way beyond what we currently know. Currently, we know very little about the prevalence and rhythm of various sensory events in everyday life. This simultaneously licenses an exploratory mindset, in which some insights are better than no insights so that an empirical foundation can take shape over time, as well as strategic considerations of what could make for the highest yield insights upon recording or annotating infants' everyday ecologies. Central to these considerations is the multi-scale nature of time. Though sampling decisions are often posed as decisions about a single timescale—“Should I record 1 h, 1 day, 1 week, 1 month, or 1 year? Should I annotate all minutes or just some minutes of each recording?”—the reality is that briefer timescales are always nested within more extended timescales and attention, memory, and learning mechanisms operate over multiple timescales as infants build knowledge (Thelen and Smith, 1994). Thus, because theories of experience-dependent change require evidence from multiple timescales of everyday experiences, it is productive to consider the extent to which any sampling decision yields a “multi-scale dividend” by potentiating insights at multiple theory-relevant timescales.

Theorists designing sampling plans face a classic conundrum in that the prevalence of their target behavior constrains optimal sampling yet prevalence itself is often unknown (Collins and Graham, 2002; Adolph et al., 2008). Importantly, prevalence at one timescale does not straightforwardly predict prevalence at other timescales. For example, individual instances of many behaviors like bouts of walking, attention to objects, and music often last on the order of seconds (Adolph et al., 2012; Suarez-Rivera et al., 2019; Mendoza and Fausey, 2021a,b) and these brief instances do not arise at a steady rate across an hour or across a day. One clear illustration of this is a pattern of speech interleaved with extended periods of silence in samples of everyday audio (Tamis-LeMonda et al., 2017; Cristia et al., 2021). That is, speech rate was not constant but rather rose and fell over the course of the extended recording. Non-uniform temporal rhythms make the endeavor of identifying a rate in a shorter sample and then linearly extrapolating to estimate its rate over longer timescales potentially suspect. Relatedly, interpolating between coarsely timed samples yields trajectories that are meaningfully distorted compared to denser sampling (Adolph et al., 2008). Thus, sampling briefly (e.g., 1 min total) or sparsely (e.g., 1 min per hour) is not likely to yield a multi-scale dividend (e.g., discoveries about secondly, minutely, and hourly prevalence). In contrast, densely annotating many hours of everyday life makes it possible to discover structure at the finest-grained annotated timescale as well as every coarser timescale up to total sampled duration of extended recordings “for free” (see also Principle 3).

Of course, few practicing developmental theorists would consider densely annotating many hours of everyday life “free.” The massive investment of person-hours required for manual annotation costs time and money; the following considerations can inform sampling decisions when balancing feasibility with ambitions of a multi-scale dividend.

Although we have a lot to learn about the prevalence of everyday behaviors, existing evidence often provides some anchors. For example, time-use and retrospective surveys completed by caregivers of young children suggest broad contours of everyday rates (Hofferth and Sandberg, 2001; PSID-CDS User Guide, 2002), such as daily occurrence for music (Custodero and Johnson-Green, 2003) and weekly rhythms for some aspects of affect and sleep (Larsen and Kasimatis, 1990; Szymczak et al., 1993). Ongoing research using complementary methods like dense Ecological Momentary Assessment in which caregivers report in-the-moment snapshots of their infants' experiences over days, weeks, and months (Franchak, 2019; Kadooka et al., 2021) will also reveal the prevalence of many motor, visual, and language behaviors at extended timescales of everyday life. One's sampling scheme can respect available evidence by sampling at least as densely as known rates, and go beyond extant knowledge by combining any of several denser and/or more extended samples. When everyday prevalence is unknown or coarsely estimated, many timescales (not just the most costly) would yield multi-scale dividends to advance theories of experience-dependent change.

Recent and ongoing efforts are also teaching us about the consequences of various sampling schemes for estimating rates of everyday behaviors within extended recordings. For example, random sampling approximates rates of continuously annotated behaviors when those behaviors are medium or high base rate (Micheletti et al., 2020). Estimates for each of two available languages in everyday speech, as well as rates of adult- and child-directed speech, stabilize upon cumulating roughly 90 min of 30-s segments randomly sampled from a day (Cychosz et al., 2020b). Behaviors with low everyday base rates present the biggest challenge for sampling; erring on the side of continuous annotation is wise for initial efforts that can then inform future sampling schemes. Another productive option is to combine multiple sampling choices such as randomly selected segments together with segments of peak theory-relevant activity (Casillas et al., 2020).

A related consideration is to identify which portions of everyday experience you must quantify in order to best address your primary research question. If sensory input during waking hours is the theory-relevant experience, then samples can be scheduled according to known waking hours per day for infants of various ages (Galland et al., 2012) instead of sampling full 24-h cycles or including mid-day naps. Portions of extended recordings like episodes dense with adult speech and therefore potential social interactions (Ramírez-Esparza et al., 2014; Romeo et al., 2018), and episodes like mealtimes that provide learning opportunities for many early learned object names (Clerkin et al., 2018), are highly relevant for many theories of experience-dependent learning. Here, theorists need only be mindful of extrapolation and interpolation missteps when using such samples to inform estimates of cumulative experience (see also Montag et al., 2018). Thus, theorists can make principled decisions about sampling schemes most likely to achieve a combination of advancing theory, avoiding estimation traps, and feasibility.

Some research questions demand large quantities of everyday data that may vexingly resist attempted downsizing via shorter and/or sparser sampling. Two examples include aiming to understand temporally extended schedules *per se*, due to their hypothesized relevance for learning mechanisms related to spacing and/or sleep consolidation (e.g., Rovee-Collier, 1995; Gómez and Edgin, 2015; Vlach, 2019) and estimating extended cumulative experiences like functions relating word tokens and types in order to understand everyday lexical diversity (Montag et al., 2018). Other research questions require capturing many instances of everyday sights and sounds (e.g., objects, words, musical tunes, speaker/singer identities, etc.) in order to understand opportunities for learners to encounter repeated and varying instances within and across categories. Accumulating multiple instances often requires extended sampling because everyday behaviors may preferentially occur in particular activities (e.g., breakfast) and on particular days of the week (e.g., only on Saturday). One dramatic illustration of this is the discovery of a total of 313 instances of the word “breakfast” in 15 months of continuously transcribed adult speech (Roy et al., 2015) which works out to fewer than one instance per day. Multiple and varied instances of other behaviors can be quantified by dense annotation within daily or hourly samples (e.g., Clerkin et al., 2018; Tamis-LeMonda et al., 2018; Mendoza and Fausey, 2021a). Altogether, if the necessary volume of everyday instances is unlikely to occur all at once or if there is not yet enough known about a phenomenon to predict when it will occur at a high volume, then theorists may need to sample extended and densely in order to discover theory-relevant distributions of experience.

Sampling is fundamentally a multi-scale matter; 10 min of a morning at home cannot represent the entire life from which it was sampled, and it might not meaningfully represent the month, day, or even hour from which it was sampled. The implications of any particular everyday sample for theories of experience-dependent change will become clearer as theorists identify patterns of relative stability and change at multiple timescales of everyday experience. Measures like coefficient of variation (Anderson and Fausey, 2019), multi-scale coefficient of variation (Abney et al., 2017a), and intra-class correlations (Bolger and Laurenceau, 2013; d'Apice et al., 2019; Mikhelson et al., 2021) all yield insights about these dynamics. Recent investigations have quantified such everyday dynamics from hour-to-hour, day-to-day, and month-to-month (e.g., Fausey et al., 2016; Anderson and Fausey, 2019; d'Apice et al., 2019) and additional insights will increasingly be possible thanks to shared corpora of many hours of infants' everyday lives.

One way to cumulate insights across timescales is to design sampling schemes with extant evidence in mind, taking care to articulate how one's scheme will yield discoveries at briefer and/or more extended timescales than currently known. Another way to potentiate multi-scale insights is to densely (not sparsely) annotate many (not few) hours of everyday life so that analyses can quantify multiple coarser-than-annotated rhythms. Determining timescales of relative stability (e.g., 10 min at the beginning and end of a day may be interchangeable) and relative change (e.g., 1 month sampled at the beginning and end of a year may not be interchangeable) for everyday phenomena will also enable greater precision in relating trajectories of experiences and developmental change. Though dense sampling of extended timescales is not a unique path to insights about infants' everyday ecologies, the multi-scale dividend for such efforts is very high and thus worth pursuing for cumulative science.

### Implementation

We extended knowledge about everyday music in infancy by creating a scheme for when and how much audio to record from everyday life, as well as when and how much audio to annotate from within captured recordings. Multiple resources relevant for implementing this principle can be found in the OSF components specified in Figure 1. One illustrative file is “Silence_Praat_Loop” (https://osf.io/egmbh/), a Praat script that accomplishes a pre-processing step designed to address situations in which families occasionally turned off the LENA™ digital language processor (DLP). In order to ensure that time in each .wav file represents time elapsed during the recorded day, this script inserts silence into .wav files for the duration of any periods when the DLP had been turned off. For example, if a family recorded from 8 a.m. until 8 p.m. and they turned off the DLP from 9 a.m. to 10 a.m., then the resulting .wav file would be 11 h instead of 12 h duration. Inserting 60 min of silence starting 1 h from the beginning of the .wav file preserves continuous time in to-be-annotated files.

#### Decide When and How Much to Record

We made a theory-informed decision to sample three full days per family distributed within 1 week. Prior research suggested that caregivers would sing and/or play music daily with their infants (Custodero and Johnson-Green, 2003), but there was not yet enough known to predict when music would occur during the day. We therefore sampled densely by instructing families to record the maximum 16-h duration of the LENA™ digital language processor. We sampled multiple days per family in order to potentiate insights about the stability and variability of everyday music across multiple timescales. Three days was based on what would be feasible for families to complete (Gilkerson et al., 2015; Canault et al., 2016).

#### Decide When and How Much to Annotate

We made a theory-informed decision to densely sample the many hours of recorded life. One of our research aims was to discover the total duration of music per day in infants' lives. Because the prevalence and timing of music bouts within a day were unknown, we annotated continuously in order to detect each bout. This approach yielded 42 h of everyday music from within 467 h of everyday sounds. We also aimed to quantify the repetition and variation of features, voices, and tunes within everyday music. Prior research suggested that unique instantiations of music might occur sparsely during infants' days (Costa-Giomi and Benetti, 2017) and so continuous annotation was most likely to identify the full range of the day's musical features, voices, and tunes.

#### Decide What Not to Annotate

We made a theory-informed decision not to annotate long stretches of silence or very low-level sounds since these portions of the recordings were unlikely to contain our phenomenon of interest. Our approach for identifying and excluding these portions of the recording is generalizable to studying other auditory phenomena and consistent with pre-processing steps used in prior research (Weisleder and Fernald, 2013; Bergelson and Aslin, 2017). We jointly addressed the priorities of sampling continuous time as well as identifying and excluding extended silences from annotation. First, we inserted silence into any period of a .wav file when the LENA™ digital language processor had been turned off during the day, in order to preserve continuous time. Next, we protected families' privacy by replacing with silence any portions of a .wav file that caregivers noted as private or outside of their home. We then automatically identified sections of the .wav file that fell below a decibel threshold (−22 dB relative to the peak amplitude for that recording) for at least 3 min. This criterion was informed by previous research (Bergelson and Aslin, 2017) and verified through testing on pilot data. Finally, we manually identified any brief sounds (under 3 min) that interrupted two otherwise adjacent periods of silence (e.g., the baby sneezed while napping) as well as any extended periods (at least 10 min) of highly regular sound (e.g., a white noise machine on during the baby's nap). These pre-processing steps generated one .txt file per recording that was read into ELAN to show the start and end times of to-be-annotated sections of the recording. Pre-processing yielded roughly 270 h of to-be-annotated data, which was 0.42 of the total recorded data. This reduction was expected due to the typical duration of sleep and mix of other activities for infants in this age range (Galland et al., 2012). Overall, we integrated the realities of unknown or sparse base rates of everyday music with theory-irrelevant portions of infants' days to settle our sampling scheme of continuously annotating pre-processed everyday audio recordings.

## Principle 3: Finest-Grained Defensible Timescale of Observed Phenomena in Many Hours of Everyday Life

With continuously recorded daylong data, it is theoretically possible to quantify rhythms at every timescale from yoctosecond (i.e., one septillionth of a second) to full-day (i.e., one 24-h time period). Practically, temporal resolution is constrained in part by the device that recorded the everyday data. For example, the LENA™ digital language processor decompresses recorded sound at a resolution of 16 kHz (Ford et al., 2008), which means that milliseconds (not yoctoseconds) is the finest-grained available timescale. Beyond device sampling rates, it is widely acknowledged that “the hardest problem in dealing with time is determining the appropriate sampling intervals” (Adolph, 2019, p. 191). For example, should one manually annotate the presence or absence of music per every millisecond, second, minute, or hour throughout continuously recorded days? Most considerations point toward a principle of sampling finer- rather than coarser-grained.

Evidence about the duration of individual episodes of one's annotation target should inform decisions about which timescale(s) to manually annotate. For example, we know that consequential behaviors in many domains are brief and last on the order of milliseconds to seconds (e.g., Adolph et al., 2012; Warlaumont et al., 2014; Suarez-Rivera et al., 2019; Mendoza and Fausey, 2021a). Because instances of these brief behaviors are not uniformly available over the full duration of an extended recording, sampling much more coarsely than their individual durations may distort prevalence estimates. For example, suppose that individual music bouts persist for seconds (not hours) and manual annotation designed to detect music within a daylong recording identifies the presence or absence of music per hour. Suppose that at least one music bout occurs within every hour, yet very few bouts persist for its entire hour. Hourly annotation sampling would yield the (distorted) conclusion that music is constant throughout the day. Note that because the durations of many everyday behaviors are currently unknown, discoveries about many temporal rhythms would advance developmental theory even if it is possible to code even finer-grained. For example, everyday rhythms annotated “per minute” are much finer-grained than “per day,” “according to retrospective caregiver report,” or “per year, theorists assume.”

Relatedly, many devices sample less frequently than once per second (e.g., Mehl, 2017; Casillas et al., 2020) in order to achieve extended battery lives. Annotators may also sample more coarsely than devices, respecting properties of human perceivers' temporal resolution or rates of environmental change. For example, researchers annotated everyday egocentric visual rhythms at 1/5 Hz from recordings captured at 30 Hz in order to make initial discoveries about the prevalence of faces and hands (Fausey et al., 2016). “Down-sampling” is sometimes used to describe such schemes, yet the resulting annotations offer theorists finer-grained insights about everyday ecologies than extant evidence. The priority is not to describe every phenomenon at the finest-grained timescale of *any* observed phenomenon, but rather at a timescale that advances theory by annotating at a temporal resolution hypothesized to be theory-relevant yet currently unknown for the target behavior.

Manually annotating many hours of everyday life also creates opportunities to discover relationships among multiple timescales. The reality of multiple nested timescales minimizes pressure to pick “the” “right” timescale because any single timescale is limited in its explanatory value for developmental change when considered in isolation (Thelen and Smith, 1994; Spencer et al., 2011). Insights about multi-scale structure could arise by aggregating across distinct investigations, or it could be the goal of a single annotation effort. For example, Allan Factor captures hierarchical clustering and can be quantified by annotating at a finer grain and then aggregating across increasingly coarser grains (Abney et al., 2017b; Falk and Kello, 2017). Recent tutorials provide theorists with additional inspiration and considerations about structure at multiple timescales of everyday experiences (Xu et al., 2020).

Generally, it is possible to aggregate from finer- to coarser- timescales without additional annotation, but not the reverse (Adolph, 2019). Finer-grained annotations also make for everyday datasets that are maximally useful as training and evaluation sets for developing automated algorithms to detect everyday behaviors (e.g., Räsänen et al., 2019). Theorists therefore maximize potential for insights for themselves, and for others upon sharing their annotations, by annotating the finest-grained defensible timescale (see also Principle 2). One constraint that places a bound on the finest-grained defensible timescale is inter-rater reliability. For example, even if a phenomenon varies from 1 millisecond to the next, two annotators may reach similar descriptions only at the timescale of seconds. Designing increasingly laborious annotator training procedures in attempts to achieve reliable finer-grained annotations often yields diminishing returns and so is not feasible (see Principle 4). Other feasibility constraints like personnel time can be managed by strategically structuring multiple passes of annotating everyday data. For example, musical features, voices, and tunes are nested within music bouts (Principle 1). By first annotating temporal onsets and offsets of music bouts at a finer-grained timescale, subsequent passes of judging the presence/absence of features (e.g., “vocal”) and identities (e.g., “Itsy Bitsy Spider”) per bout can yield temporal information without annotators having to also spend person hours marking onsets and offsets of the features and identities. Thus, theorists can optimize a suite of theoretical and practical considerations to annotate the finest-grained defensible timescale of their target phenomena in many hours of everyday life.

### Implementation

Figure 1 shows the OSF components with multiple resources relevant for implementing this principle. One illustrative file is “1_MusicBouts_SecMidnight.R” (https://osf.io/cr2mt), which smooths everyday music annotations from native ELAN milliseconds into seconds.

#### Annotating Music Bouts at the Milliseconds Timescale

Little was known about the duration of individual instances of music in infants' everyday ecologies, so we lacked robust empirical evidence to motivate a timescale for detecting everyday music bouts. We initially annotated pilot recordings at the 5-min timescale, following related manual annotation schemes for efficiently sweeping through many hours of everyday life (e.g., Weisleder and Fernald, 2013). These efforts readily revealed that everyday music bouts were much briefer than 5 min. Thus, we capitalized on ELAN's native timescale of milliseconds for continuously annotating audio recordings.

#### Smoothing Annotated Music Bouts to the Seconds Timescale

We smoothed ELAN annotations to the seconds timescale for two reasons. First, some evidence suggested that infants would encounter playsongs and lullabies (Trehub and Schellenberg, 1995; Trehub and Trainor, 1998) whose composed renditions last for seconds not milliseconds (e.g., a typical rendition of “Itsy Bitsy Spider” takes ~17 s). Second, though ELAN's default timescale is milliseconds, we did not train annotators to obsess about millisecond precision in music bout onsets and offsets which would have required listening and re-listening with unclear payoff for initial discoveries. Thus, we smoothed the atheoretical native resolutions of LENA™ and ELAN to a timescale of our observed phenomenon.

To format music bouts data into the seconds timescale, we exported the annotated data from ELAN with one row per music bout indicating its onset and offset times in milliseconds and seconds. We inclusively rounded the ELAN onset and offset times to the nearest second. We expanded the ELAN data into a timeseries of seconds starting at 0 (midnight) and continuing for 129,600 s (i.e., a 36-h time span), to achieve a shared dataframe across recordings that accommodated a small number of recordings that were recorded later in the day. We populated each second (row) in which an annotator identified music with a “1” and the remaining with “0.” If two consecutive music bouts were separated by <1 s as annotated in ELAN, then they were merged into one music bout in this timeseries. In this way, each annotator's data were transformed into a common format: a .csv file with 129,600 rows representing each second in a 36-h period starting at midnight of the recorded day. We analyzed everyday music at this timescale of seconds.

#### Merging Annotations of Musical Features, Voices, and Tunes

In subsequent annotation passes, annotators identified the features, voices, and tunes in each music bout (*N* = 4,798 bouts). These additional annotations were per bout, obviating any need for further timescale operationalizations.

Features, voices, and tunes were originally annotated per music bout. Annotators listened to each previously identified music bout using ELAN and recorded their new annotations in Excel (i.e., one row per music bout with columns for features, voices, and tunes; see OSF for additional details, https://osf.io/qjpux/). These annotations were then cleaned (e.g., removed punctuation, checked for typos) and any internal inconsistencies were remedied (e.g., a music bout annotated as “vocal” but without a voice identity; see Mendoza and Fausey, 2021a). Voice and tune identities were then replaced with de-identified labels (e.g., VoiceID1, VoiceID2) in order to protect the confidentiality of participating families.

Annotations were merged into the seconds timeseries. All seconds within a bout inherited any feature, voice, or tune identified within that bout. The disadvantage of this scheme was potential imprecision for bout-internal durations for bouts that had multiple musical features (e.g., “live” and “recorded”), voices (e.g., “Beyoncé” and “Daniel Tiger”), and/or tunes (e.g., “Old MacDonald Had a Farm” and “I'm a Little Teapot”). The advantage of this scheme was savings in person hours (Supplementary Table 5, OSF, https://osf.io/htx57/).

We discovered that many musical bouts were characterized by a single feature, voice, and tune thus yielding straightforward duration estimates. For discoveries based on estimates derived in part from bouts with multiple features, voices, and/or tunes, we conducted more conservative and more liberal analyses. We discovered similar distributional structure whether we analyzed bouts with only a single feature, voice, and/or tune or analyzed all data that potentially overestimated some feature, voice, and/or tune durations (Mendoza and Fausey, 2021a).

## Principle 4: Transparent Rationale for Assessing Reliability of Annotations in Many Hours of Everyday Life

Multiple annotations of the same everyday data should point to the same conclusion about its structure. Considerations for assessing reliability like the kind of variable, study design, and assignment of annotators to samples are relevant when annotating many hours of everyday life. Scaling from practices established using smaller and differently structured datasets, however, sometimes presents challenges with non-obvious solutions. Here, we share a mindset for grappling with these issues and point readers to other resources for specific metrics and calculations (e.g., House et al., 1981; Bakeman and Gottman, 1997; Hallgren, 2012).

Attempting to establish reliability when measuring new constructs, at new timescales, and in immense quantities may raise the blood pressure of theorists accustomed to traditionally short and sanitized behaviors captured in laboratory contexts. In the relatively more wild everyday context, it can be challenging to determine what kind and degree of reliability is “good enough.” As with other efforts at the edge of innovation, theorists should not let the perfect be the enemy of the good. Theorists can integrate extant knowledge with newly encountered realities in order to make a case for productive solutions. Innovation does not license a measurement free-for-all, but rather raises the value of showing due diligence, situating one's contribution, and transparently sharing each step of the process. Transparency is especially valuable so that other theorists can re-use, aggregate, and over time update practices as new consensus emerges.

The metrics used to assess inter-rater reliability, as well as the proportions of recorded data that are annotated in order to assess reliability, vary widely across empirical endeavors. Theorists may struggle to align reliability habits from literatures with which they are familiar to realities of their everyday data. For example, extended timescales often yield low base rates of target behaviors (e.g., many more seconds without than with music in a 16-h everyday audio recording) as well as distributional details that rarely arise at shorter timescales (e.g., it is hard to smoosh a day's 51 distinct tunes into a traditional 5-min laboratory visit). Theorists should therefore engage in due diligence in order to understand the space of available approaches to assess reliability, particularly with respect to related kinds and timescales of everyday data, and share a summary of their review. We illustrate one example of this process in our Supplementary Table 3 (OSF, https://osf.io/htx57/), which is a review of 32 papers and approaches for assessing reliability of manual annotations of some form of children's unstructured activity. Such a review is not designed to be exhaustive, but rather helpful in combating failures of imagination about potential metrics, practices, and acceptable thresholds for inter-rater reliability in many hours of everyday data.

We flag two properties of everyday data that often rise in salience as theorists consider reliability and can prompt clarity and revision to other aspects of an overall manual annotation workflow. First, because nobody re-lives the same second, minute, or hour all day long, data from many hours of everyday life include periods of activity and periods of inactivity. For many infants, naps may be one source of relatively silent periods within a day. Other rising and falling rhythms of target behaviors, due in part to the changing activities of the day (Bruner, 1975; Roy et al., 2015; Montag et al., 2018), can yield low base rates of target behaviors at a daily timescale. Should theorists include or exclude periods of inactivity in their reliability assessments, and does it depend on the source and/or temporal extent of inactivity? This issue is a construct and sampling issue rather than a reliability issue *per se*. Theorists must articulate the extent to which they aim to discover structure that includes naps; if they aim to quantify structure only in infants' waking hours, then periods of naps should not be annotated at all. Similarly, extended periods outside the home can yield acoustic properties that are distinct from most other periods of a day and could therefore be outside the scope of one's central discoveries. Second, reliably annotating everyday data becomes increasingly challenging at ever finer-grained timescales. Pilot annotation efforts that reveal unreliable annotation at one timescale often make a coarser timescale the most defensible (see also Principle 3). Is it still worth it to identify structure at coarser timescales, particularly if this diverges from typical quantifications of related behaviors sampled in more constrained contexts? As noted above, the answer is often yes. In many domains, discoveries of even hourly everyday rhythms would advance knowledge beyond current understanding and also guide future waves of inquiry. Altogether, it is productive to center contributions to developmental theory rather than prioritizing practices (often established in contexts of “high base rates reliably coded at the millisecond timescale”) that may not scale to the everyday context.

Another source of potential indecision on the way to reliable manual annotation of many hours of everyday life is establishing the quantitative threshold for “good enough.” In the absence of formal consensus, transparency is the way forward. Three strategies to arrive at an achievable and productive reliability threshold include (1) identify typical ranges of reliability via systematic review of related everyday phenomena, (2) identify the current state of algorithm-human concordance, and (3) identify the set (if not all) of captured data that can be reliably annotated.

Systematic review of related evidence (as in Supplementary Table 3, OSF, https://osf.io/htx57/) calibrates typical ranges of reliability. The achievable reliability ceiling in everyday data may be lower than in laboratory data due to lower signal-to-noise ratios arising for various reasons. Systematic reviews are themselves publishable as incremental contributions to growing literatures. Another strategy to help calibrate one's reliability threshold is to identify concordance between commonly accepted algorithmic estimates and human annotation (e.g., Cristia et al., 2021). If one's human-human annotation concordance exceeds algorithm-human concordance, then one's annotation scheme ranks favorably compared to insights based on algorithmically detected patterns. Finally, theorists can plan to analyze only those portions of their data that are reliably annotated. Multiple annotators can judge individual episodes of everyday behavior, and then only those episodes that are annotated identically by multiple annotators are analyzed (e.g., Fausey et al., 2016; Cychosz et al., 2021). With this approach, one need not drop an entire project because some of the data are difficult to annotate and contribute to a low “overall” reliability. The resulting reliably annotated dataset is often orders of magnitude larger and more diverse than other data available to advance developmental theory. Note that if the bulk of the data are difficult to reliably annotate, then theorists should revisit Principle 1 in order to design an annotation scheme that is better matched to everyday instantiations of their construct. Overall, theorists can transparently situate their contribution as “good enough” with respect to extant knowledge about their target phenomenon.

Two further dimensions of assessing reliability when annotating many hours of everyday life lead theorists to confront tension between scientific rigor and daunting personnel effort. First, should annotators identify everyday behaviors by continuously listening to or watching recordings, or could they instead annotate pre-segmented clips? Second, should multiple people annotate all data, or could some smaller proportion of the data be submitted to reliability computations? Continuous annotation is necessary when one's primary research question is to discover the durations of everyday behaviors. Continuous annotation can also make for higher reliability when one's goal is to detect repetitions of like kind (e.g., the same tune sung in the morning and in the afternoon) by maintaining available context cues from a particular family. Tools like ELAN and Datavyu make continuous coding reproducible. Under certain sampling or signal-to-noise scenarios (see Principle 2 and above), pre-segmented clips are justified and efficient. Full, and not partial, reliability is most productive when implementing an annotation scheme for the first time or in a very new context (e.g., everyday music, Mendoza and Fausey, 2021a). When annotating behaviors with wide consensus about their operationalization (e.g., utterances, words), partial reliability suffices. For partial reliability protocols, best practice is to annotate partial datafiles rather than partial datasets (e.g., 20% of each infant's recording instead of 20% of recordings; Adolph et al., 2019).

From rationale to implementation, transparency has never been easier. Increasingly, systematic reviews and meta-analyses are available (e.g., Ganek and Eriks-Brophy, 2018). Sharing one's own due diligence is straightforward (e.g., Open Science Framework). Visualizations of data together with figure captions that highlight relevant reliability can also be helpful in bridging expertises across scholarly communities (e.g., Figure 3 in Mendoza and Fausey, 2021a). Taking advantage of shared protocols can also save theorists from reinventing every aspect of a workflow (e.g., Adolph et al., 2019; Soderstrom et al., 2021). Over time, as more theorists tackle annotating many hours of everyday life in order to advance theories of developmental change, new consensus will emerge. Each theorist contributes to this consensus by making their rationale for assessing reliability transparent.

### Implementation

Figure 1 shows the OSF components with multiple resources relevant for implementing this principle. One illustrative file is “3_IRR_Tunes_Part2.R” (https://osf.io/jgw57/), which is used to calculate Tschuprow's *T* for assessing contingency between multiple annotators' distributional structure of everyday musical tunes.

As mentioned, we reviewed relevant literature in order to calibrate our approach to reliability in the new endeavor of quantifying theory-relevant properties of everyday music in infancy and we shared this review (Supplementary Table 3, OSF, https://osf.io/htx57/). Annotators continuously annotated daylong recordings, skipping silent portions (Principle 2), and so reliability computations considered these annotations. Because this was the first time anyone had quantified music and its features, voices, and tunes in many hours of everyday life, each annotation pass of each recording was fully annotated by two independent annotators. We calculated a Pearson correlation coefficient to assess reliability of annotated music bouts. For each annotated musical feature, we calculated proportion agreement at the level of music bouts. For the annotated voice and tune identities, we calculated Tschuprow's *T*, because this metric allowed us to compare two sets of nominal data with potentially different numbers of unique categories in each set of manual annotations (e.g., if Annotator 1 listed 26 unique voices and Annotator 2 listed 23 unique voices). We determined Tschuprow's *T* at the level of music bouts for annotated voice identities and tune identities. For all of these metrics, we used a reliability threshold of 0.90 because this was squarely within the range of previously reported values. Inter-rater reliability was high for all annotations (Mendoza and Fausey, 2021a). To facilitate cumulative science, we shared our data, our scripts for computing reliability, and detailed instructions about our reliability procedure (OSF).

## Principle 5: Active and Representative Training Protocol for Annotating Many Hours of Everyday Life

Every annotator must learn the detailed procedure for manual annotation and execute it reliably. The challenge, then, is how to train initially naïve annotators. Traditionally, scholars have lacked robust guiding information about how to design a training protocol for reliably annotating a complex phenomenon in many hours of everyday life. Encouragingly, this is rapidly changing and we contribute some further resources here (Casillas et al., 2017; Adolph et al., 2019; Soderstrom et al., 2021; see also Ramírez-Esparza et al., 2014; Belardi et al., 2017).

The task of manually annotating the full duration of a daylong recording requires annotators to maintain a very high level of attention to detail across many, many hours of work. Any training protocol must successfully prepare and evaluate annotators for this challenge. The principle, then, is to create an active and representative training protocol. A first phase that emphasizes active learning serves to train general skills, with annotators actively practicing generating annotations, making predictions, and asking questions. Annotators are then evaluated on their ability to annotate recordings that match the real data in both total duration and content in a second phase designed to reveal annotators' potential lapses in attention and memory across many hours.

### Implementation

Figure 1 shows the OSF components with multiple resources relevant for implementing this principle. One illustrative file is “1_MusicBouts_Coding Training_Script.pdf” (https://osf.io/xd85u/), which shows how to conduct a training session for annotators learning to annotate music bouts.

#### Offer Comprehensive Training Sessions

In our procedure, trainees actively participated in two separate training sessions, led by an expert annotator: a 1-h session on how to annotate music bouts and a 2- to 3-h session on how to annotate musical features, voices, and tunes. For each, we started with a brief overview of the study, to help trainees understand what they were listening to and why. Then, we explained the training goal: if their annotations of a full-length training recording matched at least 0.90 with those of an expert annotator, then they would be considered a “trained annotator” and they could annotate real data. Several key features of these training sessions encouraged active learning: (1) Trainees got hands-on practice navigating the server that hosts the data, setting up annotation files, and creating annotations. This was helpful because trainees' prior computer use varied widely. (2) Trainees reviewed each slide of the coding manual and listened to every audio example. We asked them to generate their own ideas about why each example was included. This, in combination with feedback from the expert annotator, helped trainees learn what they were supposed to annotate. (3) We created step-by-step instructions with screenshots for how to complete every step of the annotation process. These “how-to” documents are rich sources of information for annotators that reduced their cognitive load for completing this work. (4) We provided explicit instructions about taking breaks, working independently, and not multitasking, boosting annotators' ability to maintain high-quality work across many hours. (5) We encouraged trainees to ask questions throughout this training session. We aimed to make it clear that this type of work requires high attention to detail and also that we would provide them with a lot of support.

#### Design Representative Training Recordings

We used six daylong recordings from pilot data as training recordings. An expert annotator identified music bouts in all six recordings and then annotated features, voices, and tunes in the music bouts of three of these recordings (Supplementary Table 4, OSF, https://osf.io/htx57/). We designed training recordings that contained a range of targets that annotators would need to identify. The three recordings for annotating music bouts each had multiple instances of music, a mix of sounds that were easier and harder to identify as music, and musical sounds that occurred in one or multiple music bouts. The three recordings for annotating features, voices, and tunes each contained a wide range of musical voices and tunes in all combinations of features and included music bouts with multiple voices and/or multiple tunes.

#### Require Trainees to Annotate and Meet Criterion on a Representative Training Recording Prior to Annotating Real Data

The basic skills needed to identify music bouts and to annotate features, voices, and tunes in a 5-min recording are the same as those necessary for annotating a daylong audio recording; the challenge is endurance. We required trainees to practice annotating a full-length training recording in order to assess the extent to which they could maintain high-quality annotating across the entire duration of a daylong audio recording. Trainees annotated separate recordings for music bouts and for musical features, voices, and tunes. We used the same criteria and procedures for assessing reliability as described in Principle 4, with trainees' annotations compared to the expert's annotations. If a trainee failed to reach the 0.90 criterion on their first training recording, then they received feedback, practice, and further training. They could then annotate up to two additional training recordings. If they failed to reach criterion on all three training recordings per annotation pass, then they never annotated real data for this project. Roughly three-quarters of trainees met criterion on their first recording, with a handful achieving criterion after two or three recordings. Occasionally, a trainee failed to reach criterion and/or decided to stop working on this project prior to completing the training.

## Principle 6: Procedural Priority on Selective Attention When Annotating Many Hours of Everyday Life

In daylong audio recordings of everyday environments, there is a lot to notice in the complexity of real life (Xu et al., 2009). Annotators are tasked with identifying a specific phenomenon, such as music, among a mix of many everyday sounds, including people talking, siblings laughing, dogs barking, dishwashers running, and more. This task presents several challenges. Annotators may encounter multiple, varying forms of a complex phenomenon of interest. Annotators may need to use lots of information in order to identify the phenomenon, such as detailed definitions and multiple audio examples of which sounds should and should not be annotated as music. It may not be possible for annotators to learn and remember all forms of the phenomenon in advance. For example, no single annotator could be expected to recognize every tune and every artist from every genre of music on the radio. With so much information to keep in mind, annotators' attention may drift both in the moment and also over time across a long-term project. The solution to these challenges is to build in practices that support annotators' attention and memory. Thus, designing a procedure that prioritizes selective attention is the principle. Researchers can boost annotators' attention and memory by including (1) distinct annotation passes for annotating one well-defined annotation target at a time, (2) regular review of annotation targets as well as options for searching for and creating annotation labels, and (3) routine quality assurance checks. These aspects of an annotation procedure reduce the challenges of annotating complex phenomena in many hours of everyday life.

### Implementation

Figure 1 shows the OSF components with multiple resources relevant for implementing this principle. One illustrative file is “5_MusicBouts_CheckUpClips_InstructionsToCoders.pdf” (https://osf.io/cn3ke/), which shows one example of step-by-step instructions to annotators as well as check-up clips used for routine quality assurance checks.

#### Include Distinct Annotation Passes for Distinct Annotation Targets

To reduce the challenge of identifying the many forms of music, we implemented five separate annotation passes, each with one distinct target: music bouts, live and/or recorded features, vocal and/or instrumental features, voice identities, and tune identities. This procedure prioritized selective attention by requiring annotators to focus on one well-defined annotation target (i.e., one property of music) at a time, thereby minimizing the cognitive burden for annotators.

#### Require Regular Review of Annotation Targets

We provided annotators with manuals that contained a lot of information and audio examples for identifying the annotation targets of each pass (Principle 1). To boost annotators' memory for this information, annotators reviewed the relevant section of the manual for their current pass at the beginning of each work session. This helped annotators to keep the definitions and examples of music, features, voices, or tunes fresh in their minds. It also helped them to transition from whatever activity they were doing before their work session into the annotation task, thus enhancing selective attention and minimizing divided attention (e.g., sending e-mails and/or working on coursework). Annotators could also return to the manual at any point during their work sessions, which further reduced the amount of information they needed to hold in their working memory while annotating.

#### Allow Searching for and/or Inventing Labels

There are clear limits to annotators' knowledge of and memory for all potential forms of music and musical features, voices, and tunes. For example, an annotator might recognize a radio voice as Beyoncé but not know the name of the tune. Our procedure included two elements that enabled annotators to increase their own knowledge of the many forms of music: (1) Annotators completed a one-time media review prior to annotating the data for musical features, voices, and tunes. This review familiarized annotators with the wide range of musical sounds likely to occur in infants' everyday environments (i.e., TV shows, music, and toys created for children and for adults). It also reminded them that they would likely hear musical sounds from sources they have not personally encountered before (e.g., a children's TV show that they had not seen) and that they should still strive to identify the specific voices and tunes therein. Note that the examples in this media review were from Western culture, intentionally selected from the cultural context in which the participating families lived. (2) Annotators used the internet to search for voice and tune identities when they heard a musical sound that they could not immediately recognize. They were not allowed to use any song-identifying software (e.g., Shazam) that would directly access the raw audio recordings (i.e., confidential data). They were also not allowed to consult any human resource since this could violate the independence of their annotation and/or compromise data confidentiality. In addition to searching for existing labels, annotators were allowed to invent their own open-ended labels for voice and tune identities if they could not determine the specific identity for standard tunes (e.g., “upbeat pop song”) or if someone in the recording invented a tune on the spot (e.g., “parent's toes song”). License to invent labels helped annotators avoid perseverating on never-ending searching or second-guessing and released attention to tackle subsequent annotations.

#### Build in Routine Quality Assurance Checks

Across a project, annotators might pay less attention to the detailed definitions for music and musical features, voices, and tunes. For example, an annotator could at some point start to judge a parent's vocal car sound effects as music, even though these kinds of sound effects are explicitly listed as not music in the manual. To avoid this kind of attentional drift, annotators completed routine quality assurance checks. These checks consisted of manually annotating one “check-up clip” after every two daylong audio recordings annotated. For music bouts, each check-up clip was either one 20-min segment or two 10-min segments selected from pilot audio recordings. The expert annotator manually annotated each check-up clip. We compared the trained annotator's manual annotations with those of the expert annotator, using the same procedures for assessing agreement as for the full training recordings (Principle 4), with one exception. Because the duration of check-up clips was short, any single agreement or disagreement (that could be random) carried more weight. So, we adjusted the check-up agreement criterion from *r* = 0.90 to *r* = 0.80. If annotators met this agreement criterion, then they resumed annotating real-data audio recordings. If not, then they were given up to two more check-up clips to annotate. If their annotations of the second or third check-up clip met the agreement criteria, then they returned to annotating real data. If they did not reach the agreement criteria on any of the three check-up clips, then they did not annotate any further real data and their annotations for their two most recently annotated recordings were replaced. We implemented the same check-up clip procedure for manually annotating features, voices, and tunes. These check-up clips each consisted of 10 music bouts selected from pilot recordings annotated by the expert annotator. We used the same agreement criteria as for the full training recordings and the same logic for determining if an annotator should continue to annotate real-data recordings. Overall, no annotator's manual annotation drifted to the point that they were removed from the project.

## Principle 7: Inclusive Physical, Social, and Professional Communities for Annotators of Many Hours of Everyday Life

Manually annotating many hours of everyday life is not an easy task. It takes a lot of time. Annotators spend long hours working at their computer stations. The bulk of the work must be done independently, so annotators may feel isolated or like they are a cog in a machine. Annotators must sustain high levels of focus in order to detect specific targets that may occur infrequently. This makes the task simultaneously cognitively demanding and boring. The key challenge is to maintain motivation among annotators so that they continue to generate high-quality annotations throughout a long-term project. Creating a healthy community is the principle. Recruiting a large, diverse group of annotators creates an inclusive community. Encouraging annotators to work as a team helps them develop a sense of belonging and feel invested in the work. Providing opportunities for annotators to build skills and to receive mentorship from senior colleagues adds to the value for annotators, keeping them engaged in the work. Adding in fun activities recognizes annotators' humanity, increasing their enthusiasm to be actively involved. Setting up a physical workspace with varied ways to work comfortably makes annotators ergonomically happy. Annotators who work as part of inclusive physical, social, and professional communities are more likely to stay and to do high-quality work. Avoiding high team turnover is especially important when training procedures require investing roughly 25 h per person.

### Implementation

Figure 1 shows the OSF components with multiple resources relevant for implementing this principle. One illustrative file is “2_LearningLab_Bingo_Winter2018.png” (https://osf.io/x4gd8/), which is one example of a lab practice designed to promote community.

#### Recruit a Large and Diverse Team

**Figure 2:**
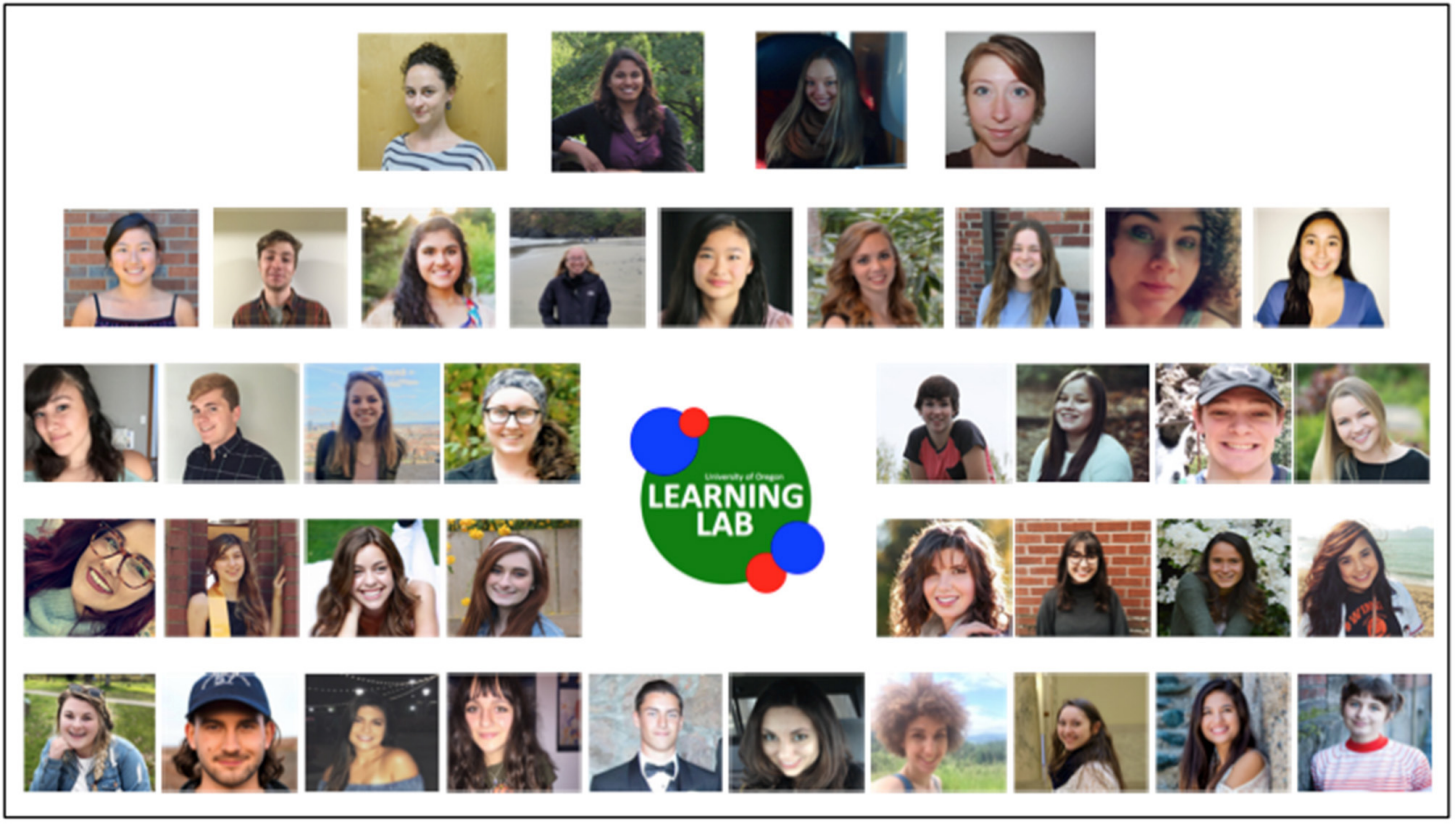
Photos of UO Learning Lab team members who manually annotated music and musical features, voices, and tunes, including (top row, left to right): Dr. Caitlin Fausey (PI), Dr. Jennifer Mendoza (doctoral student at the time), Catherine Diercks (lab manager), Christine White (lab manager), and 35 research assistants (left to right, Row 2: Hitomi Tanizawa, Josh Mabry, Vinitha Gadiraju, Emma Salmon, Adeline Fecker, Helen Rawlins, Madison Edgar, Sabrina Haskinson, Kayla Figone, Row 3: Aiko Luckasavage, Samuel Hickman, Melissa Lattig, Erin Batali, Katie Mickel, Sophie Cohen, Thorin Faulk, Jennifer Lowery, Row 4: Jayne Coles, Cayla Lussier, Amanda Powell, Kyra Wilson, Jordyn Mons, Grace Floyd, Juliette Tisseur, Arie Markowitz, Row 5: Brittany Brann, Mitchell Passadore, Allysia Rainey, Natalie Draga, Liam Green, Melissa Berg, Kelly Woltjer, Rachel Ward, Jewel Montes, Keelan Paroissien-Arce).

In the University of Oregon Learning Lab, we do not require undergraduate students to be psychology majors, to have research experience, or to have completed specific coursework prior to applying for a research assistant (RA) position. These practices reduce some systemic barriers for institutionally underrepresented students in academia to become directly involved in research, actively promoting equity and inclusion. For this project, we also did not require students to have prior formal or informal musical training. Our team of music annotators (Figure 2) had varied majors, including psychology, music, computer science, physics, sociology, linguistics, and human physiology. Their music experience ranged from none to lifelong. We found this mix to be beneficial because they collectively provided a wide range of insights and observations. Manual annotation research combined with our approach to building a team is well-suited to diversifying the scientific workforce.

#### Meet Often in Varied Configurations

Our team actively participates in multiple weekly lab meetings, each designed to advance our scientific research and to promote professional development. Meeting with varied configurations of lab personnel (in person or virtually) provides opportunities for lab members to build different skills. For this project, annotators attended project team meetings, led by Mendoza, where they discussed the ongoing music annotation work, asked questions, and shared observations about the data (limiting specifics to preserve annotator independence). They built skills for project management by collectively reviewing progress, setting concrete goals, and prioritizing weekly tasks. During our full-lab meeting, all lab personnel participated in a mix of science-skill-building activities, including discussing empirical studies, giving elevator pitches about our work, and using statistical computing tools (e.g., R & Python). RAs steered these meetings, voicing their ideas and questions. Lastly, during small senior personnel meetings, Fausey met with graduate students, lab managers, and select senior RAs. Trainees took the lead, asking questions, collectively problem solving, and soliciting feedback about their research, thus supporting senior personnel to develop more advanced science skills. By holding each of these different meetings on a weekly basis, we lowered barriers to identifying problems and accelerated finding solutions.

#### Include Activities That Recognize the Humanity of the Team

To build a sense of community, we regularly include tasks designed to humanize the experience of working in a research lab. At the beginning of each meeting, everyone shares a fun fact about themselves. When large teams regularly use the lab space, Mendoza and Fausey frequently work in the lab, intentionally creating opportunities to talk with lab personnel. Each term we play lab bingo, with bingo cards filled with science tasks (e.g., made a plot, asked a question, used R, called a family) and we have bingo prizes. We make up science raps and songs, both for entertainment and to boost our learning. Having fun and being part of a community is motivating; lab personnel gain the sense that they matter, that their work matters, and that their work affects other people in the lab.

#### Design a Physical Workspace to Promote Well-Being

We created a workspace that allowed for many RAs to work simultaneously so they would not feel isolated. We had computers dedicated for annotators to use while annotating music data. We also maintained a large assortment of chairs (including yoga balls) and an adjustable standing desk for annotators to use. This mix of furniture helped keep lab personnel ergonomically happy and minimized the potential for repetitive stress injuries from working long hours at a computer (Tompa et al., 2010). Lonely annotators who are in physical pain will generate low-quality work and ultimately leave. Thus, creating a physically and socially inclusive work environment was critical to the success of our annotation team.

Using these multiple strategies, we created an inclusive physical, social, and professional community and provided lab personnel with rich educational experiences. Our healthy, positive working environment supported annotators to conduct an estimated total of 6,400 h of manual annotation (Supplementary Table 5, OSF, https://osf.io/htx57/). Annotators understood that we valued their contributions to the team. They also recognized that we were supporting their professional development. In addition to direct experience conducting research, they gained knowledge and skills that would help prepare them for a wide range of future positions both within and outside developmental science.

## Discussion

Insights into infants' everyday ecologies are increasingly available to developmental theorists thanks to the combination of wearable technologies to record these ecologies (de Barbaro, 2019), infrastructure to share the everyday recordings (MacWhinney, 2000; VanDam et al., 2016; Gilmore et al., 2018), and protocols to facilitate detecting structure in these everyday samples (Adolph et al., 2019; Soderstrom et al., 2021). Rigorously quantifying everyday ecologies advances theories of developmental change by centering tasks, timescales, and trajectories that are not discoverable in traditional laboratory protocols (Dahl, 2017; Rogoff et al., 2018; Smith et al., 2018; Frankenhuis et al., 2019; de Barbaro and Fausey, in press). One exciting and daunting frontier is to quantify everyday opportunities for learners to attend, encode, retrieve, and integrate experiences over not just one but many timescales. Here, we articulated a framework with an eye toward optimizing multi-scale dividends upon investing considerable resources in manually annotating many hours of everyday life. We encourage theorists to jump into the endeavor of quantifying everyday ecologies in order to make discoveries about the experiences upon which development may depend.

Importantly, everyday ecologies vary across the world's communities. Cross-cultural variation is evident in infants' opportunities to encounter child-directed speech (Casillas et al., 2020), sing with caregivers (Trehub and Schellenberg, 1995), move and explore (Rachwani et al., 2020), and more. Multiple levels of context organize experiences (Rowe and Weisleder, 2020); quantifying everyday ecologies across variation in these contexts will advance theories about multiple pathways of developmental change. This ambitious goal is attainable in part by annotating existing corpora that span the world's cultures (e.g., Benetti and Costa-Giomi, 2019; Bergelson et al., 2019) as well as mindfully sampling and annotating more everyday ecologies over time. We highlight that Principles 1 and 6 may prove especially helpful across distinct annotation endeavors, with research teams investing effort in iterative pilot annotations in order to arrive at constructs that are meaningful within specific communities as well as training annotators with representative recordings.

Relatedly, accelerating the breadth and pace of discoveries is also more likely with an ever more diverse and inclusive community of scholars. Manually annotating existing corpora is one research activity that is amenable to contributions across researchers who have varying expertises, working environments, cultural contexts, and resources. Such diversity is also deeply necessary in order to minimize biases in operationalizing everyday behaviors as they arise in many contexts (e.g., Cychosz et al., 2020a). Aggregating contributions from many individuals and teams means that smaller efforts cumulate to larger insights, making team science well-matched to the challenge of annotating many hours of infants' everyday lives (see also Cychosz et al., 2021). Frameworks designed to address issues that arise in research that is distributed over time and teams include co-authorship and contributorship models (Holcombe, 2019; Moshontz et al., 2021), pre-registration of secondary data analyses (Van den Akker et al., 2019), and protocols devised for widespread use coupled with practical tutorials to support incremental contributions (Soderstrom et al., 2021).

Resources like HomeBank (VanDam et al., 2016), Databrary (Gilmore et al., 2018), and Open Science Framework (https://osf.io/) are vital to maintain and expand because they make it possible for theorists to transparently make progress collectively. These are living repositories, potentiating new discoveries about human development through curation of more and different data over time. Notably, multiple funding agencies helped launch these repositories and dedicate specific grant mechanisms to support secondary data analyses at multiple scales (e.g., NIH R03, NSF SBE HNDS-I). Continued investment and diverse engagement will maximize the value of these collective treasures and propel developmental science forward.

Overall, contributions of many kinds will be necessary to build a diverse and cumulative science of everyday ecologies. For discussion of practical tradeoffs facing any individual theorist—including rapid vs. delayed theoretical gratification, going it alone vs. collaborating, and sampling selectively vs. exhaustively—see de Barbaro and Fausey (in press). One sign of productive manual annotations at scale will be its demise after theorists have used annotated everyday datasets to successfully train algorithms to automatically detect theory-relevant behaviors in the hubbub of everyday sights, sounds, and more. We are currently very far from this goal in most domains and there may be no surer way out than through. Manually annotating many hours of infants' everyday lives is likely to spur innovation not only in theories of developmental change but also in the tools used for future discoveries.

Quantifying everyday ecologies can inspire theorists to pursue hypotheses that might not arise from other sources (see also Nastase et al., 2020). For example, instead of presenting learners with input distributions inspired by traditional laboratory instantiations of consistent amounts of multiple category instances distributed evenly over time, learning theorists might instead appreciate the striking prevalence of non-uniform content and temporal distributions in everyday ecologies (e.g., Smith et al., 2018; Mendoza and Fausey, 2021a,b) and test hypotheses about the consequences of these distributions (e.g., Casenhiser and Goldberg, 2005; Carvalho et al., 2021). Manipulating training regimes for both human learners and models, with parameters shown to be plausible in everyday ecologies, will bring developmental theory closer to meeting longstanding goals of jointly modeling the input and its impact (e.g., Smith and Slone, 2017). Quantifying distributions of everyday parameters encountered across learners will also inspire new routes to understanding individualized developmental pathways by combating failures of imagination due to traditional one-size-fits-all training protocols (e.g., Thelen and Smith, 1994; Samuelson, 2021). All of these potentially dramatic expansions to future hypothesis testing are within reach of any theorist making use of everyday corpora.

The current moment in developmental science is full of opportunities for game-changing discoveries by taking advantage of methods that scale beyond traditional laboratory experiments. For example, developmental theorists can now implement experiments beyond the reach of only their local community (e.g., ManyBabies, Frank et al., 2017; Lookit, Scott and Schulz, 2017). New tools enable theorists to aggregate across large bodies of evidence (e.g., MetaLab, Bergmann et al., 2018). Quantifying everyday ecologies similarly scales beyond traditional contexts and timescales available to ground theories of development. The framework presented here can support theorists as they embark on efforts to annotate many hours of infants' lives en route to discovering more about the experiences available to drive experience-dependent change.

## Data Availability Statement

In accordance with family consent, audio recordings and extracted music clips are available on HomeBank (doi: 10.21415/T5JM4R; doi: 10.21415/T47D-5K51). Study materials, behavioral coding manuals, numerical data, and analysis code are available on Open Science Framework (doi: 10.17605/osf.io/eb9pw).

## Ethics Statement

The studies involving human participants were reviewed and approved by Institutional Review Board, University of Oregon. Written informed consent to participate in this study was provided by the participants' legal guardian/next of kin. Written informed consent was obtained from the individual(s) for the publication of any potentially identifiable images or data included in this article.

## Author Contributions

JM and CF contributed to all aspects of manuscript preparation. All authors contributed to the article and approved the submitted version.

## Supplementary Material

The Supplementary Material for this article can be found online at: https://osf.io/eb9pw/

## Conflict of Interest

The authors declare that the research was conducted in the absence of any commercial or financial relationships that could be construed as a potential conflict of interest.

## Publisher's Note

All claims expressed in this article are solely those of the authors and do not necessarily represent those of their affiliated organizations, or those of the publisher, the editors and the reviewers. Any product that may be evaluated in this article, or claim that may be made by its manufacturer, is not guaranteed or endorsed by the publisher.
